# New Coleoptera records from New Brunswick, Canada: Gyrinidae, Carabidae, and Dytiscidae

**DOI:** 10.3897/zookeys.179.2582

**Published:** 2012-04-04

**Authors:** Reginald P. Webster, Ian DeMerchant

**Affiliations:** 1 Natural Resources Canada, Canadian Forest Service - Atlantic Forestry Centre, 1350 Regent St., P.O. Box 4000, Fredericton, NB, Canada E3B 5P7

**Keywords:** Gyrinidae, Carabidae, Dytiscidae, new records, Canada, New Brunswick

## Abstract

*Dineutus assimilis* Kirby and *Dineutus discolor* Aubé of the Family Gyrinidae are newly reported from New Brunswick, Canada. Four species of Carabidae, *Agonum (Agonum) piceolum* (LeConte), *Bembidion (Pseudoperyphus) rufotinctum* Chaudoir, *Harpalus (Harpalus) opacipennis* (Haldeman), and *Pterostichus (Melanius) castor* Goulet & Bousquet are newly reported from New Brunswick and the Maritime provinces, and one species of Dytiscidae, *Liodessus noviaffinis* Miller, is newly recorded for the province. Collection, habitat data, and distribution maps are presented for each species.

## Introduction

This paper treats new species records from New Brunswick, Canada of the Coleoptera families Gyrinidae, Carabidae, and Dytiscidae. The fauna of these families from New Brunswick and the Maritime provinces was recently treated by Majka and Kenner (2009) (Gyrinidae), Webster and Bousquet (2008) (Carabidae), and Webster (2008) (Dytiscidae). Intensive sampling in New Brunswick by the first author has yielded additional new provincial records in the above families. The purpose of this paper is to report on these new records. A brief synopsis of each family is included in the results.

## Methods and conventions

The following records are based on specimens collected during a general survey by the first author to document the Coleoptera fauna of New Brunswick.

### Collection methods

Various methods were employed to collect the specimens reported in this study, and these are included in the bionomic notes accompanying each species. A description of the habitat was recorded for all specimens collected during this survey. Locality and habitat data are presented exactly as on labels for each record. This information is summarized and discussed in collection and habitat data section for each species.

### Specimen preparation

Males of some species were dissected to confirm their identity. The genital structures were dehydrated in absolute alcohol and either mounted in Canada balsam on celluloid microslides or glued onto cards and then pinned with the specimens from which they originated.

### Distribution

Distribution maps, created using ArcMap and ArcGIS, are presented for each species in New Brunswick. Every species is cited with current distribution in Canada and Alaska, using abbreviations for the state, provinces, and territories. New records for New Brunswick are indicated in bold under Distribution in Canada and Alaska. The following abbreviations are used in the text:

**Table:** 

**AK**	Alaska	**MB**	Manitoba
**YT**	Yukon Territory	**ON**	Ontario
**NT**	Northwest Territories	**QC**	Quebec
**NU**	Nunavut	**NB**	New Brunswick
**BC**	British Columbia	**PE**	Prince Edward Island
**AB**	Alberta	**NS**	Nova Scotia
**SK**	Saskatchewan	**NF & LB**	Newfoundland and Labrador*

*Newfoundland and Labrador are each treated separately under the current Distribution in Canada and Alaska.

Acronyms of collections examined or where specimens reside referred to in this study are as follows:

**AFC** Atlantic Forestry Centre, Natural Resources Canada, Canadian Forest Service, Fredericton, New Brunswick, Canada

**CNC** Canadian National Collection of Insects, Arachnids and Nematodes, Agriculture and Agri-Food Canada, Ottawa, Ontario, Canada

**NBM** New Brunswick Museum, Saint John, New Brunswick, Canada

**RWC** Reginald P. Webster Collection, Charters Settlement, New Brunswick, Canada

## Results

### Species accounts

All records below are species newly recorded for New Brunswick, Canada. Species followed by ** are newly recorded from the Maritime provinces of Canada.

### Family Gyrinidae Latreille, 1810

A general overview of the Gyrinidae (the whirligig beetles) of North America was provided by Roughley (2001). Gyrinids are often observed in aggregations on the surface of the water and swim rapidly in circles when disturbed. Adults occur in both lentic and lotic habitats and are scavengers and predators of small insects on the water surface (Roughley 2001). Larvae are predaceous on aquatic insect larvae and nymphs (Oygur and Wolfe 1991; Roughley 2001). Majka and Kenner (2009) reviewed the gyrinid fauna of the Maritime provinces of Canada and reported 17 species from New Brunswick, including four species newly reported for the province. Recent survey work by the first author has resulted in the discovery of two additional species from New Brunswick. See Majka and Kenner (2009) for a list of the other species occurring in the province.

### Subfamily Gyrininae Latreille, 1810

#### Dineutus assimilis

Kirby, 1837

http://species-id.net/wiki/Dineutus_assimilis

Map 1

##### Material examined.

**New Brunswick, Carleton Co.**, Juniper Station in the Juniper Barren, 46.5538°N, 67.1840°W, 21.VI.2005, R. P. Webster, black spruce / tamarack bog, margin of pond (2, RWC). **Gloucester Co.**, off Hwy 8 near Allardville, 47.4303°N, 65.5163°W, 25.VI.2005, R. P. Webster, black spruce bog, on margin of small shallow pond with emergent grasses (1, RWC). **Queens Co.**, *ca*. 3.5 km W of Lower Gagetown, 45.7497°N, 66.1846°W, 13.V.2008, R. P. Webster, old red oak and red maple forest, in small pond (1, RWC); Cranberry Lake P.N.A. [Protected Natural Area], 46.1125°N, 65.6075°W, 28.VII.2009, R. Webster & M.-A. Giguère, old red oak forest, u.v. light (1, AFC). **York Co.**, Canterbury, Browns Mtn. Fen complex, 45.8841°N, 67.6428°W, 8.VI.2004, D. Sabine & R. Webster, sedge marsh in small pond (1, RWC); Charters Settlement, 45.8395°N, 66.7391°W, 23.VII.2007, R. P. Webster, mixed forest, u.v. light (1, RWC).

**Map 1.**
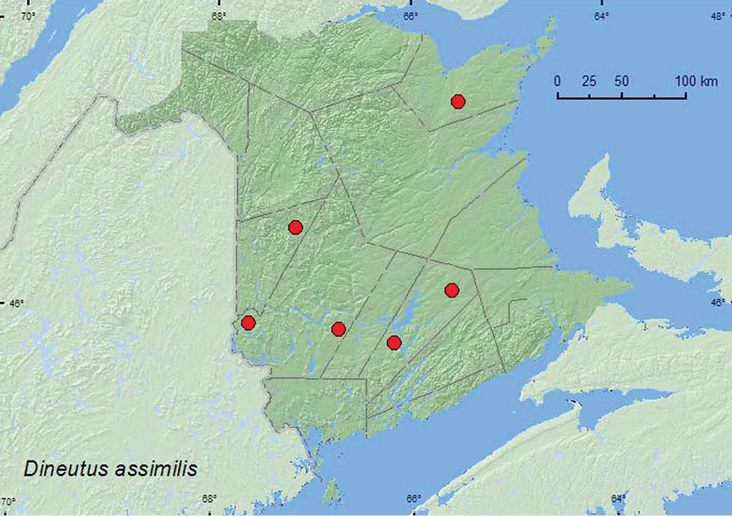
Collection localities in New Brunswick, Canada of *Dineutus assimilis*.

##### Collection and habitat data.

In New Brunswick, *Dineutus assimilis* was collected along pond margins with scattered emergent vegetation in black spruce (*Picea mariana* (Mill.) BSP) and tamarack (*Larix laricina* (Du Roi) Koch.) bogs, in a small pond in a *Carex* marsh, and in a small pond on the margin of a red oak (*Quercus rubra* L.) and red maple (*Acer rubrum* L.) forest. A few individuals were collected at ultraviolet light. Adults were captured during May, June, and July.

##### Distribution in Canada and Alaska.

BC, AB, SK, MB, ON, QC, **NB**, PE (Roughley 1991).

#### Dineutus discolor

Aubé, 1838

http://species-id.net/wiki/Dineutus_discolor

Map 2

##### Material examined.

**New Brunswick, Carleton Co.**, Jackson Falls, 46.2257°N, 67.7437°W, 12.IX.2009, R. P. Webster (river margin) (1, RWC). **Sunbury Co.**, Juvenile Settlement at S. Branch of Oromocto River, 45.5341°N, 66.6096°W, 27.VI.2006, M.-A. Giguère & R. Webster (4, RWC). **York Co.**, Fredericton, Rt. 105 at Nashwaaksis River, 45.9850°N, 66.6900°W, R. P. Webster, 28.VI.2005, 6.V.2006, river margin in embayment with sand gravel bottom, sun-exposed (5, RWC).

**Map 2.**
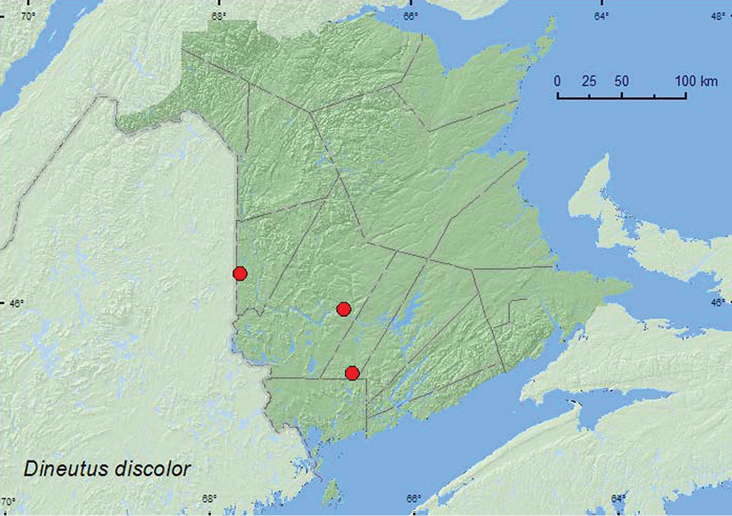
Collection localities in New Brunswick, Canada of *Dineutus discolor*.

##### Collection and habitat data.

In Quebec, *Dineutus discolor* was usually found in clear, running water (Morrisette 1979). New Brunswick specimens were collected along river margins in embayments. Adults were collected during May, June, and September in New Brunswick.

##### Distribution in Canada and Alaska.

ON, QC, **NB**, NS (Roughley 1991).

### Family Carabidae Latreille, 1802

The Carabidae (the ground beetles) is a large family with 2,635 species and subspecies in North America (Ball and Bousquet 2001). Ball and Bousquet (2001) provided a general review of the Carabidae of North America with keys to genera and information on the distribution and bionomics of the North American genera. Later, Larochelle and Larivière (2003) summarized the known natural history and biology of the North American species of this family. Most recently, Bousquet (2010) provided an illustrated key to the adults and larvae of the ground beetles of northeastern North America. These works should be consulted for details on the taxonomy, natural history, and biology of members of this family. Many Carabidae are predators on arthropods or scavengers of dead or dying arthropods; others are predators on seeds (Ball and Bousquet 2001; Larochelle and Larivière 2003). Carabids are usually ground dwellers, as their common name implies, although some species are arboreal and live under bark, on trunks or branches, or are associated with vegetation. Many species are hygrophilous or periaquatic, occupying marshes, swamps forests, riparian zones, and other damp habitats. Other species are xerophiles and live in dry forests, grasslands, and sandy habitats (Ball and Bousquet 2001; Larochelle and Larivière 2003). Webster and Bousquet (2008) provided an overview of the Carabidae of New Brunswick and reported 50 species new to the province, bringing the total number of species known from the province to 328. Recent survey work by the first author has resulted in the discovery of four additional species from New Brunswick, all of which are new to the Maritime provinces. See Webster and Bousquet (2008) and Bousquet (2010) for a list of the other species known from New Brunswick.

### Subfamily Trechinae Bonelli, 1810

#### Bembidion (Pseudoperyphus) rufotinctum

Chaudoir, 1868**

http://species-id.net/wiki/Bembidion_rufotinctum

Map 3

##### Material examined.

**New Brunswick, Carleton Co.**, Jackson Falls, 46.2257°N, 67.7437°W, 12.IX.2009, 22.V.2010, R. P. Webster, river margin above waterfall, on exposed bedrock (23, CNC, NBM, RWC).

**Map 3.**
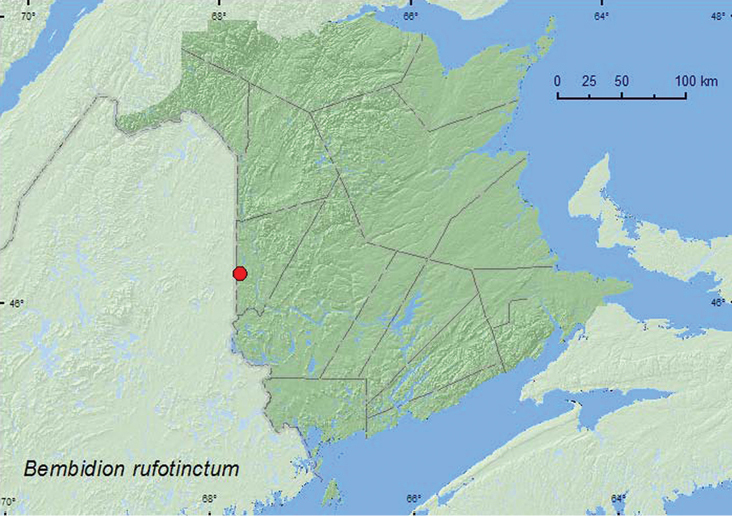
Collection localities in New Brunswick, Canada of *Bembidion rufotinctum*.

##### Collection and habitat data.

*Bembidion rufotinctum* lives in cracks of emergent bedrock in river channels (Cooper 1976; Davidson 1981). A typical habitat for this species is illustrated by Maddison (2008; Fig. 1 D, p. 148). In New Brunswick, adults were collected during May and September by splashing exposed bed rock adjacent to fast-flowing water above a waterfall. It took 5–10 min. before the adults appeared on the exposed rock surfaces. The exposed bedrock at this site was similar to that illustrated by Maddison (2008), although less extensive.

##### Distribution in Canada and Alaska.

QC, **NB** (Bousquet 1991; Maddison 2008). The closest localities of *Bembidion rufotinctum* to New Brunswick are in Quebec, Ste.-Raphaël, Bellechasse Co., on exposed bedrock near waterfalls along the Rivière du Sud (Webster, unpublished), and in New Hampshire (Maddison 2008). *Bembidion rufotinctum* has not yet been reported from Maine (Majka et al. 2011) but will undoubtedly be found in the state once appropriate habitats are sampled.

### Subfamily Harpalinae Bonelli, 1810

#### Harpalus (Harpalus) opacipennis

(Haldeman, 1843)**

http://species-id.net/wiki/Harpalus_opacipennis

Map 4

##### Material examined.

**New Brunswick, York Co.**, Queensbury, 12.VIII.1998, (G. Gesner & J. Sweeney) pitfall trap (2, AFC).

**Map 4.**
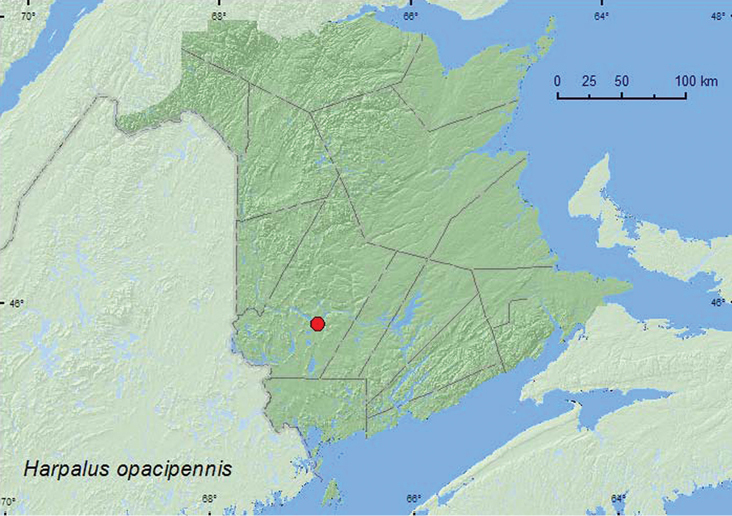
Collection localities in New Brunswick, Canada of *Harpalus opacipennis*.

##### Collection and habitat data.

There were no habitat data associated with the specimens. The site where adults were collected was a cone and seed orchard with dry meadow vegetation among trees. The two specimens were captured in a pitfall trap during August. This species is usually associated with dry habitats with sand or gravel soils with sparse vegetation, including gravel and sand pits, vacant fields, meadows, and clearings (Lindroth 1968; Bousquet 2010).

##### Distribution in Canada and Alaska.

AK, YK, NT, BC, AB, SK, MB, ON, QC, **NB** (Bousquet 1991).

#### Pterostichus (Melanius) castor

Goulet & Bousquet, 1983**

http://species-id.net/wiki/Pterostichus_castor

Map 5

##### Material examined.

**New Brunswick, York Co.**, Fredericton, 45.9361°N, 66.6747°W, 17.VIII.2009, R. Webster, D. McAlpine & G. Forbes, beaver lodge, within wall of lodge (one adult was teneral) (2, NBM, RWC); Charters Settlement, 45.8456°N, 66.7267°W, 1.V.2010, R. P. Webster, beaver lodge, under large branches on surface of lodge (10, NBM, RWC).

**Map 5.**
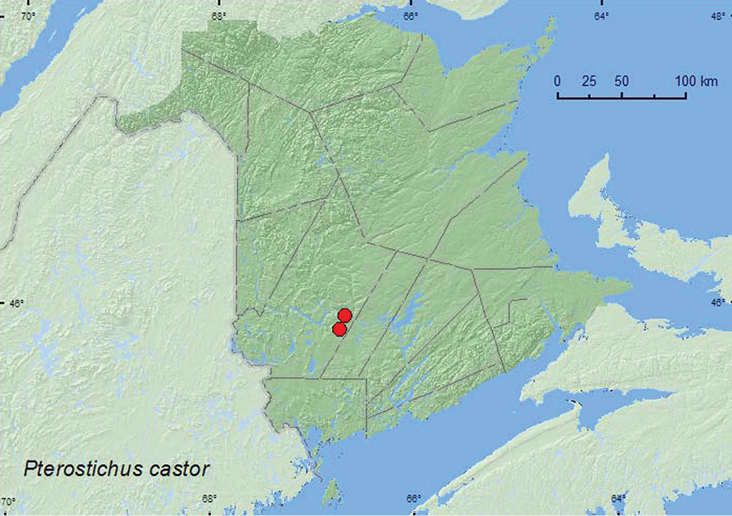
Collection localities in New Brunswick, Canada of *Pterostichus castor*.

##### Collection and habitat data.

Specimens collected in early May were found under large branches on the surface of an North American beaver (*Castor canadensis* Kuhl.) lodge, on the southwest-facing side of the lodge, on a sunny, warm day. Adults were common (over 20 observed), and one mating pair was observed, suggesting that adults may move to the surface of the lodge during the spring for mating. *Pterostichus castor* lives exclusively in inhabited or recently deserted beaver lodges or houses (Goulet and Bousquet 1983; Bousquet 1998; Larochelle and Larivière 2003).

##### Distribution in Canada and Alaska.

ON, QC, **NB** (Bouquet 1991).

#### Agonum (Agonum) piceolum

(LeConte, 1879)**

http://species-id.net/wiki/Agonum_piceolum

Map 6

##### Material examined.

**New Brunswick,** **Madawaska Co.**, Gagné Brook at First Lake Rd., 47.6077°N, 68.2534°W, 23.VI.2010, M. Turgeon & R. Webster, northern hardwood forest, shaded brook among gravel on gravel bar, splashing and turning gravel (2, RWC). **Restigouche Co.**, Jacquet River Gorge P.N.A., 47.8066°N, 66.0911°W, 13.VIII.2010, R. P. Webster, eastern white cedar & balsam fir forest, shaded brook, gravel bar, splashing gravel (2, NBM, RWC).

**Map 6.**
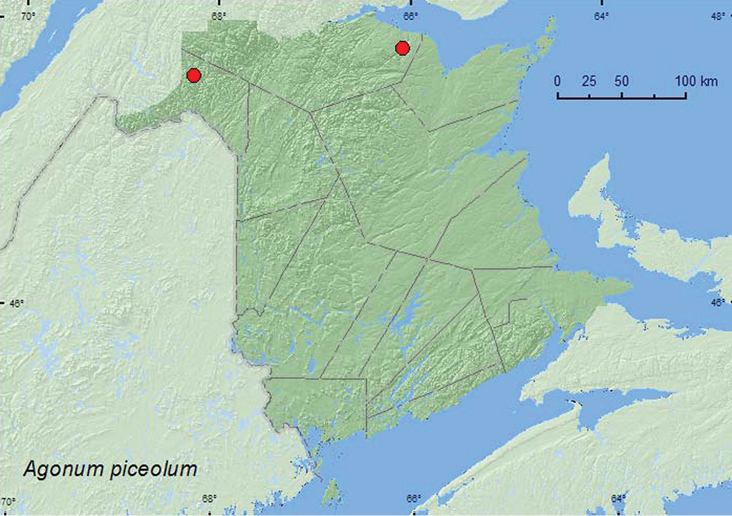
Collection localities in New Brunswick, Canada of *Agonum piceolum*.

##### Collection and habitat data.

*Agonum piceolum* is a northern transcontinental species usually found near rivers, streams, and lake shores in shaded areas, often among dead leaves on sparsely vegetated soil (Lindroth 1966; Larochelle and Larivière 2003). Specimens from New Brunswick were collected from gravel on gravel bars along cold, shaded brooks in a northern hardwood forest and an eastern white cedar (*Thuja occidentalis* L.) and balsam fir (*Abies balsamea* (L.) Mill.) forest. Adults were collected during June and August.

##### Distribution in Canada and Alaska.

BC, AB, SK, MB, ON, QC, **NB**, NF (Bousquet 1991).

### Family Dytiscidae Leach, 1815

The family Dytiscidae (predaceous diving beetles) of Canada and Alaska was reviewed by Larson et al. (2000). Species of Dytiscidae, as their common name implies, are predaceous (and scavengers) and aquatic and occur in a variety of a aquatic habitats, including small ponds, lake and stream margins, vernal ponds, springs and seeps, and even in saturated moss (Larson et al. 2000). Webster (2008) reviewed the Dytiscidae of New Brunswick and reported 18 species new for the province, including *Hydrocolous filiolus* (Fall), which was new to Canada, bringing the total number of species known from the province to 104. Here, we report another species that is new for New Brunswick. See Webster (2008) for a list of the other species occurring in the province.

### Subfamily Hydroporinae Aubé, 1836

#### Liodessus noviaffinis

Miller, 1998

http://species-id.net/wiki/Liodessus_noviaffinis

Map 7

##### Material examined.

**New Brunswick, Saint John Co.**, 45.1182°N, 67.3790°W, 28.V.2010, R. P. Webster, salt marsh, saline tidal pond (23, NBM, RWC).

**Map 7.**
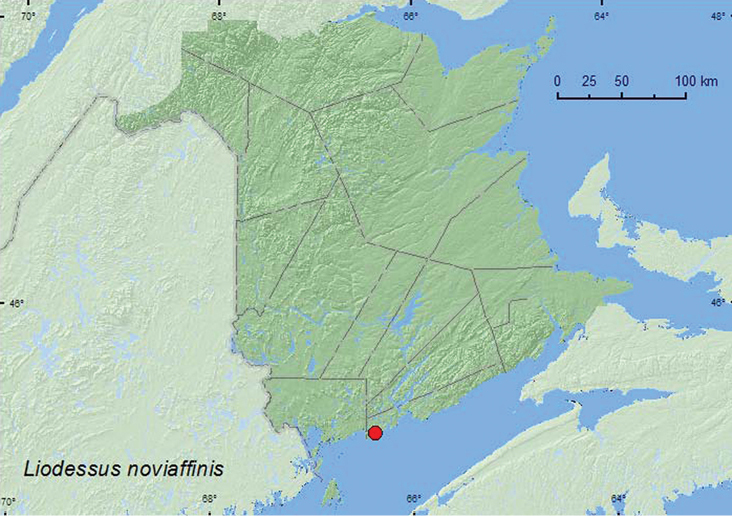
Collection localities in New Brunswick, Canada of *Liodessus noviaffinis*.

##### Collection and habitat data.

Miller (1998) reported that this species occurs in coastal ponds and may be somewhat halophilic. The specimens from New Brunswick were collected during late May from saline tidal ponds and pools near the margin of a salt marsh. Adults were abundant, along with numerous salt marsh mosquito larvae.

##### Distribution in Canada and Alaska.

**NB**, NS (Larson et al. 2000). The determination was based on dissected individuals. This species was previously known from Canada from coastal habitats on Cape Breton Island, Nova Scotia (Larson et al. 2000).

## Supplementary Material

XML Treatment for Dineutus assimilis

XML Treatment for Dineutus discolor

XML Treatment for Bembidion (Pseudoperyphus) rufotinctum

XML Treatment for Harpalus (Harpalus) opacipennis

XML Treatment for Pterostichus (Melanius) castor

XML Treatment for Agonum (Agonum) piceolum

XML Treatment for Liodessus noviaffinis
